# Common transcriptional programs and the role of chemokine (C-C motif) ligand 20 (*CCL20*) in cell migration of cholangiocarcinoma

**DOI:** 10.17179/excli2019-1893

**Published:** 2020-01-20

**Authors:** Hay Mar Win Maung, Waraporn Chan-On, Nawapol Kunkeaw, Prasong Khaenam

**Affiliations:** 1 Center for Standardization and Product Validation, Faculty of Medical Technology, Mahidol University, Nakhon Pathom, 73170, Thailand; 2 Center for Research and Innovation, Faculty of Medical Technology, Mahidol University, Nakhon Pathom, 73170, Thailand; 3 Institute of Molecular Biosciences, Mahidol University, Nakhon Pathom, 73170, Thailand

**Keywords:** CCL20, CCR6, cholangiocarcinoma (CCA), microarray, epithelial-mesenchymal transition (EMT), cell migration

## Abstract

The incidence of cholangiocarcinoma (CCA) has risen in many countries, but there is still no appropriate screening and treatment available. The growing number of microarray data published todays can be a powerful resource for the discovery of biomarkers to tackle challenges in the management of CCA. This study analyzed multiple microarray datasets to identify the common transcriptional networks in CCA and select a possible biomarker for functional study in CCA cell lines. A systematic searching identified 4 microarray datasets from Gene Expression Omnibus (GEO) repository and PubMed articles. Differential expression analysis between tumor and normal tissues was performed in each dataset. In order to characterize the common expression pattern, differentially expressed genes (DEGs) from all datasets were combined and visualized by hierarchical clustering and heatmap. Gene enrichment analysis performed in each cluster revealed that over-expressed DEGs were enriched in cell cycle, cell migration and response to cytokines while under-expressed DEGs were enriched in metabolic processes such as oxidation-reduction, lipid, and drug. To explain tumor characteristics, genes enriched in cell migration and response to cytokines were further investigated. Among these genes, CCL20 was selected for functional study because its role has never been studied in CCA. Moreover, its signaling may be regulated by disrupting its only receptor, CCR6. Treatment with recombinant CCL20 induced higher cell migration and increased expression of N-cad. In contrast, knockdown of CCR6 by siRNA reduced cell migration ability and decreased N-cadherin level. Altogether, these results suggested the contribution of CCL20/CCR6 signaling in cell migration through epithelial-mesenchymal transition process. Thus, CCL20/CCR6 signaling might be a target for the management of CCA.

## Introduction

Cholangiocarcinoma (CCA), the primary cancer of bile duct epithelium, has a high incidence rate in Thailand but is rare in other parts of the world (Kirstein and Vogel, 2016[12]). Interestingly, its incidence in many parts of the world has increased (Khan et al., 2019[11]; Patel and Benipal, 2019[20]). Chronic infection with the liver fluke, *Opisthorchis viverrini*, is the major risk factor for the development of CCA in northeast Thailand (Sripa et al., 2007[25]). Abnormal growth and the accumulation of genetic and epigenetic aberrations eventually result in malignant transformation of the bile duct epithelium (Sripa et al., 2007[25]). The morbidity and motility rate of this disease is high because of its late clinical presentation (Goral, 2017[7]). Currently, the most effective treatment is tumor resection. Nevertheless, patients with advanced cancer might not be eligible for this treatment (Wu et al., 2019[29]). Early diagnosis and a more effective therapeutic approach remain a major challenge for CCA.

High throughput technology, such as microarray, has been used for decades. Several biomarkers have been identified since then contributing to a better understanding and management of either physiological or pathological conditions (Butler et al., 2017[4]; Mazandu et al., 2017[17]). Continuous improvement in bioinformatics enables scientists to gain more insight from their data (Mack et al., 2014[15]). Compared to the classical research design that focuses on evaluating a particular set of genes or molecules, a data-driven and hypothesis-free method analyzes all possible targets available in the sample to elucidate the global mechanism of the disease and facilitate the less biased approach for biomarker discovery.

Given the availability of microarray data from Gene Expression Omnibus (GEO) database, this work revisited and selected differentially expressed genes (DEGs) from multiple microarray datasets. Chemokine (C-C motif) ligand 20 (CCL20), which was over-expressed in CCA and enriched in cell migration and response to cytokine networks, was selected for functional study. Its role has been demonstrated in many cancers including breast cancer and colorectal cancer (Xu and Pasche, 2007[30]; Marsigliante et al., 2016[16]). Its contribution to epithelial-mesenchymal transition (EMT) process, a major mechanism of cancer migration, has been also demonstrated (Katsuno et al., 2013[10]; Hou et al., 2015[8]). However, the role of CCL20 in CCA remains unknown. Thus, this work examined the expression of CCL20 and its receptor, CCR6, and investigated their function in CCA cell lines.

## Materials and Methods

### Selection of microarray dataset

**Figure 1:**
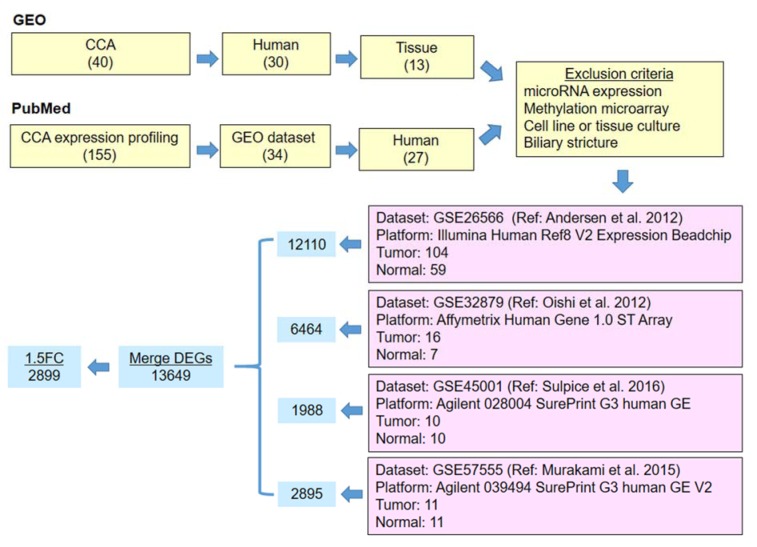
Selection of microarray datasets and identification of DEGs. Datasets in GEO and PubMed were identified by the indicated keywords. The number of datasets identified by each keyword was shown in the corresponding parenthesis. Four datasets were selected in the final step based on the exclusion criteria. GEO accession number, microarray platform, number of tumor and normal tissues, and the reference for each of these datasets are listed. DEGs obtained from each dataset were combined (n = 13649). DEGs that were over- or under-expressed in at least 3 datasets (n = 2899) were selected for the downstream analysis.

Microarray data were identified in the GEO (https://www.ncbi.nlm.nih.gov/geo/) and PubMed (https://www.ncbi.nlm.nih.gov/pubmed/) databases on April 6, 2017 by using keywords “Cholangiocarcinoma” and “Cholangiocarcinoma AND Expression profiling”, respectively (Figure 1(Fig. 1)). Human (*Homo sapiens*) tissue sample was used as the initial inclusion criterion for search results in GEO. In PubMed, the GEO dataset accession number must be provided and microarray experiment must be performed in humans. The following exclusion criteria were used for removing the remaining datasets: miRNA expression profile, methylation microarray, cell line or tissue culture, biliary stricture samples, and the number of normal tissues was less than 10 % of the sample size.

### Microarray data analysis and gene enrichment analysis

Data were inspected and evaluated the requirement for the additional pre-processing step. If needed, pre-processing in each dataset was performed separately by using “limma” package from R/Bioconductor (Ritchie et al., 2015[21]; Zhong et al., 2018[31]). In general, the pre-processing step includes target file reading, background correction, data normalization, removal of control and low quality probes, and log2 transformation were performed. The data sets GSE26566, GSE32879, GSE45001, and GSE57555 retained 22182, 18308, 17812 and 19624 probes respectively after pre-processing. Unsupervised analysis and outlier detection was performed by using principal component analysis (PCA). DEGs (false discovery rate; FDR <0.05) between tumor and normal tissue for each dataset were identified by using “limma” method. Fold-change (FC) was calculated between the average intensity of tumor and normal samples. Gene symbol was used to map and combine FC values from each dataset. FC of 1 was assigned for a missing value. In case of a duplicated gene symbol, the probe with higher intensity was used as the representative of that gene symbol. DEGs with FC of at least ± 1.5 in at least 3 datasets were selected and visualized by unsupervised hierarchical clustering heatmap to discover the common expression pattern across all datasets. Dendrogram was cut according to tree height. Gene network analysis was performed by STRING (https://string-db.org/). Enriched biological processes with FDR<0.05 were reviewed.

### Cell line and cell culture

Human CCA cell lines HuCCT1 and TFK-1 were purchased from the RIKEN BRC (Ibaraki, Japan). Cells were cultured in RPMI-1640 (Gibco, NY) with 10 % fetal bovine serum (FBS) (Gibco, NY) and 100 U/mL penicillin and 100 µg/mL streptomycin (Invitrogen, MA) and incubated at 37 °C in a 5 % CO_2_ humidified atmosphere.

### RNA isolation and real-time reverse transcriptase polymerase chain reaction (RT-PCR)

Total RNA was isolated using Vivantis GF-1 Total RNA Extraction Kit (Vivantis Technologies, Malaysia) according to the manufacturer's protocol. One µg of RNA was converted to cDNA with the Mastercycler Nexus GX2 (Eppendorf, Germany) using iScript cDNA Synthesis Kit (Bio-Rad, CA) according to the manufacturer's protocol. Quantitative PCR reaction was prepared using SsoFast Evagreen (Bio-Rad, CA, USA) in conjunction with specific primers as follows: *CCL20 *forward 5'-CAA CTT TGA CTG CTG TCT TGG AT-3' and reverse 3'-ACT TTT TTA CTG AGG AGA CGC AC-5', *CCR6 *forward 5'-TGC TCT ACG CTT TTA TTG GG-3' and reverse 3'-TTG TCG TTA TCT GCG GTC TC-5' and *GAPDH* forward 5'-GTG GAC CTG ACC TGC CGT CT-3' and reverse 3'-TGT CGC TGT GGG TGA GGA GG-5'. The reaction was performed through CFX96 Real-Time Thermocycler detection system (Bio-Rad, CA) including initial denaturation at 95 °C for 30 seconds, followed by 40 cycles of denaturation at 95 °C for 30 seconds and annealing/extension at 60 °C for 5 seconds. Melt curve analysis was performed at 65 °C to 95 °C. Relative quantitative expression is calculated by means of fold change (∆Ct) after normalizing with the reference gene *GAPDH. *The experiment was carried out in three independent sets.

### Knockdown of CCR6

Reverse transfection was carried out using Lipofectamine® 3000 (Thermofisher Scientific, CA) according to the manufacturer's protocol. Briefly, 15 pmol negative control siRNA (Silencer Select Negative Control No.1 siRNA, Thermo Scientific, CA) or CCR6 siRNA (siCCR6, s3214, Thermo Scientific, CA) was diluted in serum-free Opti-MEM medium (Thermo Scientific, CA). Diluted siRNA was incubated at the ambient temperature for 5 minutes before the addition of Lipofectamine® 3000. The complex was incubated at the ambient temperature for 20 minutes and transferred to cell suspended in an antibiotic-free medium. Transfected cells were incubated at 37 °C for 12-16 h until the cells were completely attached to the plate. The RPMI-1640 medium containing 5 % FBS was replenished. The transfection was incubated for 24 h until used in the subsequent experiment.

### Western blot analysis

Total protein was extracted by RIPA lysis buffer (Cell Signaling Technology, MA) containing protease inhibitor cocktail (Cell Signaling Technology, MA). Protein concentration was measured by Bradford protein assay (Bio-Rad, CA). Cell lysate in the amount of 15 µg (for all proteins) or 50 µg (for N-cad detection in TFK-1) was loaded onto 10 % sodium dodecyl sulfate-polyacrylamide gel electrophoresis and transferred onto nitrocellulose membrane (Bio-Rad, Germany). The membrane was blocked with 5 % skim milk and later probed overnight at 4 °C with primary antibodies including 1:1000 mouse anti-human CCR6 (14-1969-82, Thermo Fisher Scientific, CA), 1:10000 rabbit anti-human α-tubulin (2144, Cell Signaling Technology, MA), 1:5000 rabbit anti-human E-cadherin (3195, Cell Signaling Technology, MA), 1:5,000 rabbit anti-human N-cadherin (13116, Cell Signaling Technology, MA), 1:5,000 rabbit anti-human β-actin (4967, Cell Signaling Technology, MA). The membrane was washed 3 times and probed with appropriate secondary antibody including goat 1:20,000 anti-mouse-horseradish peroxidase (626520, Invitrogen, MA) and 1:5,000 goat anti-rabbit-horseradish peroxidase (7074, Cell Signaling Technology, MA). The chemiluminescence intensity was measured by ECL Western blotting detection (GE Healthcare, UK) under Gel Doc^TM^ XP^+ ^Gel Documentation System equipped with Image Lab^TM^ Software version 6.0.0 (Bio-Rad, CA). The experiment was carried out in three independent sets.

### Wound healing assay 

Cells seeded in 12-well plate were incubated until it reaches 90 % confluency. The cell monolayer was scratched with a yellow pipette tip. The wound area was photographed at 0, 6, 9 and 12 h under the light microscope and measured by ImageJ software version 1.8.0 (National Institutes of Health, USA). Relative wound area compared to 0 h was calculated. The experiment was carried out in three independent sets.

### Cell proliferation (MTS) assay 

HuCCT1 cells were transfected with siRNA in 96-well plate and incubated for 12 h. The medium was replaced with RPMI-1640 supplemented with 10 % FBS and further incubated for 24, 30 and 36 h followed by the addition of CellTiter 96 Aqueous One solution (Promega, WI). The reaction was incubated for 2 h and the absorbance at 490 nm was measured by Synergy HTX multi-mode reader (BioTek, VT). The assay was performed in three independent sets of triplicate wells.

### Cell migration assay

HuCCT1 cells were transfected with siRNA for 24 h before collected by trypsinization. TFK-1 were collected directly without siRNA transfection. 1.5 x 10^5^ cells were resuspended in a FBS free-RPMI-1640 and plated inside the Transwell insert (Corning Incorporated, NY). The lower chamber containing a RPMI-1640 supplemented with 5 % FBS or 5 % FBS with recombinant CCL20 (rCCL20, Thermo Fisher Scientific, CA). After 12 h (HuCCT1) or 16 h (TFK-1) incubation at 37 °C, non-migrated cells inside the upper membrane were removed by cotton swab and washed twice with phosphate buffered saline. The membrane was fixed with absolute ethanol for 10 minutes at room temperature and stained with 0.5 % crystal violet. The number of migrated cells was counted from 10 randomly selected areas. The number of migrated cells in relation to the respective control was calculated. The experiment was carried out in three independent sets.

### Statistical analysis

Statistics analysis was performed using student's *t *test from GraphPad Prism 7 (GraphPad Software, CA). The p-value of < 0.05 was considered statistically significant. 

## Results

### Over-expression of cell cycle and cell migration and under-expression of metabolic processes were common in CCA datasets 

Microarray datasets were searched and selected from both GEO repository and PubMed literature to include as much as possible all available datasets (Figure 1(Fig. 1)). After a systematic selection, 4 datasets were identified including 2 datasets from GEO searching, GSE45001 (Sulpice et al., 2016[26]) and GSE57555 (Murakami et al., 2015[18]), and 2 datasets from PubMed searching, GSE26566 (Andersen et al., 2012[1]) and GSE32879 (Oishi et al., 2012[19]). All of these microarray datasets were obtained from CCA tissues and a minimum sample size of 10 % non-tumor tissues. 

**Figure 2:**
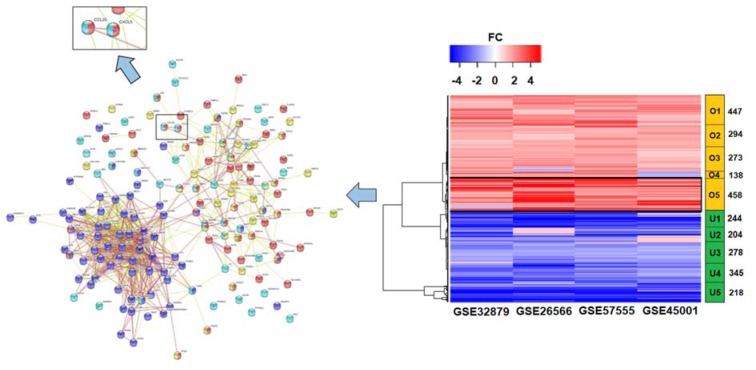
Expression profile of 2899 DEGs across 4 datasets and gene network analysis for DEGs in cluster O5. *Right panel*: Hierarchical clustering of 2899 DEGs with 1.5-fold over- or under-expressed in at least 3 datasets. Rows are DEGs and columns are datasets. Blue and red indicate under- and over-expression in CCA relative to their non-tumor tissues. Cluster ID and its number of DEGs were shown on the right of the heatmap: O, over-expressed (orange) and U, under-expressed (green) cluster. The enclosed area indicates subcluster O5, the uniformly over-expressed DEGs across all 4 datasets. *Left panel*: Gene network analysis for cluster O5. Nodes are color coded as followed: red, cell migration; cyan, response to cytokine; yellow, development of blood vessel; and blue, cell cycle. The location of CCL20 was zoomed in and shown in the square at the top of the network.

Differential expression analysis between tumor and normal tissues identified a total of 13649 unique DEGs including 1988, 2895, 12110, and 6464 from GSE45001, GSE57555, GSE26566 and GSE32879, respectively. 2899 DEGs were selected by filtering with an average fold-change of ± 1.5 in at least 3 datasets, of which 1610 were over-expressed and 1289 were under-expressed (Figure 2(Fig. 2)). The heatmap shows consistent trends in the expression of these DEGs in all datasets. As expected, DEGs were separated into clusters of over-expression (O) and under-expression (U). To aid the gene enrichment analysis, we further divided these major clusters into 5 (O1 - O5) and 5 (U1 - U5) sub-clusters, respectively (Supplementary Figure 1 and Supplementary Table 1). 

Gene enrichment analysis was performed for each subcluster. Due to a large number of significantly enriched biological processes (FDR < 0.05) and redundancy of gene ontology terms, manual reviewed was performed on enriched processes. Networks, which cover high percentage of gene list were considered the representative of enriched networks for each subcluster. After overall analysis, we found that cell cycle, cell migration, response to cytokine and blood vessel development were the common enriched biological processes for subclusters O1 to O5. While lipid metabolic process, drug metabolic process, blood coagulation and regulation of complement activation were commonly enriched for each subclusters U1 to U5 (Table 1(Tab. 1)). Based on this result, over-expressed genes might play an important role for cancer characteristics such as cell cycle and cell migration. Therefore, genes in cluster O5 were considered for subsequent analysis because of their highest magnitude of over-expression. To link to the tumor microenvironment, genes in networks of cell migration and response to cytokine were reviewed. Ten genes including *ADAM9*,* CCL20*,* COL1A1*,* COL1A2*,* CXCL5*,* FOXC1*,* LEF1*,* MIF1*,* MMP1*, and* WNT5A* were enriched in both cell migration and response to cytokine. Among these genes, the role of *CCL20* has not been studied in CCA. Moreover, this chemokine has only one specific receptor CCR6, their specific effect in CCA might be investigated by modulating their interaction. Therefore, CCL20 and CCR6 were chosen for functional validation in our study.

### Expression of CCL20/CCR6 and the EMT markers in CCA cell lines

**Figure 3:**
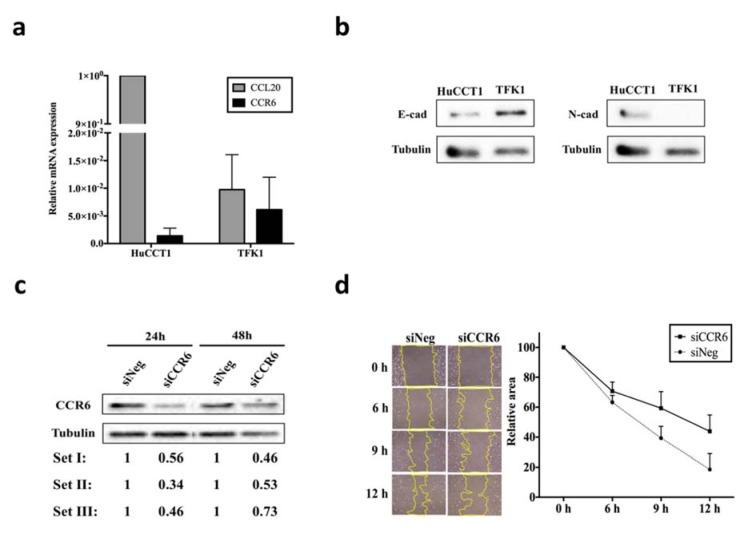
mRNA and protein expression of *CCL20* and *CCR6*, and the effect of CCR6 knockdown on wound healing ability in CCA cell lines. a) Real-time RT-PCR for *CCL20* (gray bar) and *CCR6* (black bar) in HuCCT1 and TFK-1. b) Baseline expression of E-cad and N-cad in HuCCT1 and TFK-1. c) Representative Western blot assays in 24 and 48 h siNeg and siCCR6 transfected HuCCT1. Relative protein expression from 3 independent assays was shown below the corresponding lane. d) Wound healing assay in siNeg and siCCR6 transfected HuCCT1. Image (40X) was recorded at indicated time points, the yellow line highlighted the wound closure area (left). Graph shows mean + SE for relative wound closure area from 3 independent assays in siNeg (circle) and siCCR6 (square) transfected cells (right).

To investigate the role of CCL20 and CCR6 in CCA, mRNA expression of these genes were screened in HuCCT1 and TFK-1 cells using real-time RT-PCR. As shown in Figure 3a(Fig. 3), different expression levels of *CCR6 *and *CCL20* was observed in these cell lines. Markedly high *CCL20* expression was observed in HuCCT1, while the expression of both genes was comparable in TFK-1. Next, we examined the role of CCL20 in EMT process. The constitutive expression of both E-cadherin (E-cad) and N-cadherin (N-cad) was detected in HuCCT1 with 15 μg of protein used in Western blot assay, whereas only E-cad could be detected in TFK-1 (Figure 3b(Fig. 3)). Nevertheless, it was possible to detect N-cad with 50 μg cell lysate in TFK-1 (see Figure 5c). Altogether, the results highlighted the difference in expressions of CCL20 and EMT markers in HuCCT1 and TFK-1. Constitutive expression of *CCL20* in HuCCT1 may be responsible for its higher expression of mesenchymal markers such as N-cad. To further validate the involvement of CCL20/CCR6 in EMT process, CCA cell lines were treated with siCCR6 or rCCL20 and migration assays were performed. 

### siCCR6 transfection in HuCCT1 and TFK-1

*CCR6* knockdown assays were performed in both HuCCT1 and TFK-1. Western blot analysis showed a decrease in CCR6 expression of approximately 40 % and 30 % after 24 and 48 h reverse transfection with siCCR6 in HuCCT1, respectively (Figure 3c(Fig. 3)). The knockdown efficiency in TFK-1 was < 20 % (data not shown), therefore, HuCCT1 was selected for CCR6 knockdown assay and related functional study.

### Delayed wound closure and decreased cell migration in siCCR6 transfected HuCCT1 

**Figure 4:**
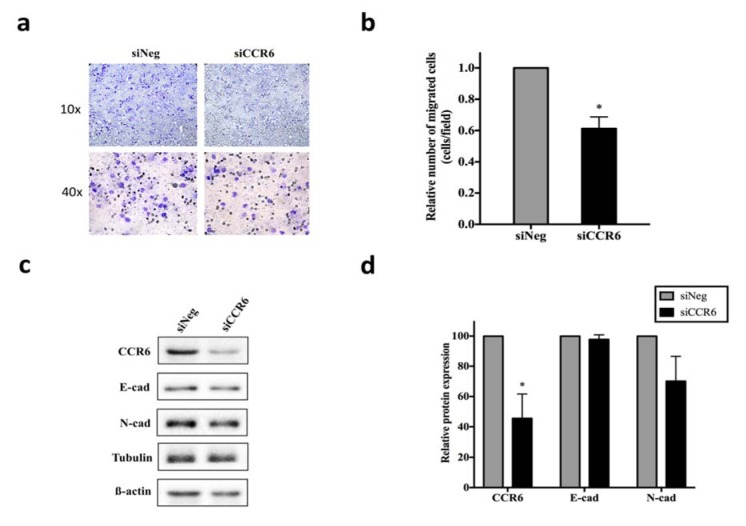
Effect of CCR6 knockdown on cell migration and EMT process in HuCCT1. a) The representative image of the transwell membrane. The relative number of migrated cells from three independent assays were shown in b), * indicates p<0.05. c) Western blot analysis for CCR6, N-cad, E-cad, α-tubulin, and β-actin. Relative protein expression from three independent Western blot assays was shown in d), * indicates p<0.05.

To examine the role of CCL20/CCR6 signaling in cell migration and proliferation, wound healing assay was performed in siCCR6 transfected HuCCT1. Compared to siNeg transfected cells, two-fold slower wound closure was observed in siCCR6 transfected HuCCT1 (Figure 3d(Fig. 3)). MTS assay was performed to determine the effect of siCCR6 on cell proliferation, there was no significant difference in proliferation rate between the siCCR6 and siNeg transfected HuCCT1 (Supplementary Figure 2). Nevertheless, we could confirm the effect of siCCR6 on cell migration ability through transwell assay in which the number of migrated cells decreased significantly (p < 0.05) compared to siNeg transfected HuCCT1 (Figure 4a and b(Fig. 4)). Therefore, the EMT markers, E-cad and N-cad, were further analyzed. Although the difference in E-cad expression was not observed, N-cad expression in siCCR6 transfected cells was slightly decreased suggesting the partial contribution of CCL20/CCR6 signaling in cell migration through EMT process (Figure 4c and d(Fig. 4)). We also analyzed the migration ability in rCCL20 treated HuCCT1, however, only slightly increased in the number of migrated cells could be observed (Supplementary Figure 3).

### rCCL20 induces cell migration through EMT process in TFK-1

**Figure 5:**
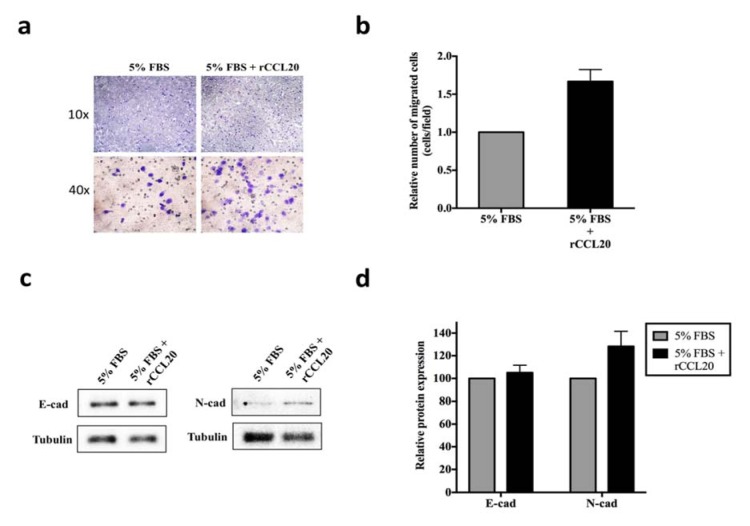
The effect of rCCL20 treatment on cell migration and EMT process in TFK-1. Cells were cultured in medium containing 5%FBS with or without 100 ng/ml rCCL20. a) The representative images at 10X (top) and 40X (bottom) magnification of the transwell membrane. The relative number of migrated cells from three independent assays were shown in b. c) Western blot analysis for E-cad, N-cad, and α-tubulin (loading control). Relative protein expression from three independent western blot assays was shown in d.

To avoid the effect of high constitutive CCL20 expression, we performed rCCL20 treatment and employed transwell assay to determine its effect on cell migration in TFK-1. Here we verified that the presence of rCCL20 in the lower chamber of the transwell plate substantially increases the number of migrated cells (Figure 5a and b(Fig. 5)). In addition, the increase in N-cad expression was observed in rCCL20 treated cells suggesting its effect on EMT process (Figure 5c and d(Fig. 5)).

## Discussion

Transcriptional profiling is an important resource for the development of precision medicine. Recent advances in technology, bioinformatics, as well as the accumulation of data in public repository lead to better insight into disease biology and eventually result in a more effective diagnosis and treatment approach. While RNA sequencing is more insightful, its number in CCA is still too small, so many integrative or meta-analyses have been performed mainly on microarray data. In this study, we not only employed a data-driven approach to identify a novel biomarker but also performed a functional validation to confirm its potential utility for CCA management. Recently Zhong et al. (2018[31]) have reported a similar integrative analysis of 7 microarray datasets. The additional 2 datasets were not included in our selection because the proportion of normal tissues in their studies was lower than 10 %. The analysis approach is another difference between Zhong's and our study. The variation in samples and technology platforms was our major concern. Accordingly, we performed differential expression analysis separately in each dataset before combining them. We then selected transcripts that show similar expression pattern across all datasets. This approach may not retain all DEGs but assure the consistency across multiple studies. To the best of our knowledge, our work is the first study demonstrating the role of CCL20/CCR6 in CCA. Nevertheless, the more comprehensive functional study in CCA is still needed because its role in other cancers is unclear and limited. 

Although, some previous studies have reported that there is decrease in cell proliferation in cancer cell lines during EMT process (Kapur et al., 2016[9]; Liu et al., 2017[13]), while as others have found that there is no effect on cell (Vongsa et al., 2009[27]; Boyle et al., 2015[3]). From the results, our finding is in accordance with later group. Such variation may be associated with time, characteristics of cell lines, and cell culture condition. 

We demonstrated the effect of CCL20/ CCR6 on cell migration, probably through EMT process, in line with many studies. The difference in the pattern of E-cad and N-cad expressions in HuCCT1 and TFK-1 led us to consider the association between EMT markers and characteristics of these cell lines. Since, HuCCT1 was derived from a metastatic CCA, we hypothesize that its condition may be more biased toward a mesenchymal state. Interestingly, we found the high constitutive expression of N-cad and low expression of E-cad in this cell line (Figure 3b(Fig. 3)). This result was consistent with a previous report in CCA (Lu et al., 2014[14]). We further confirmed this association by limiting CCL20/CCR6 signaling using siCCR6 transfection. Although it was not statistically significant, knockdown of CCR6 likely resulted in a decrease in N-cad expression. In contrast to HuCCT1, TFK-1 had lower expression of *CCL20* and N-cad but higher expression of E-cad. Treatment with rCCL20 could increase the expression of N-cad confirming the contribution of CCL20/CCR6 signaling in EMT process. However, we did not observe any changes in the expression of E-cad. Thus, it is necessary to find an optimum time point to determine changes in E-cad expression or include other markers to determine the EMT process.

Although the primary role of CCL20 is in inflammation process, it also contributes to changes in microenvironment that promotes the growth and progression of tumor cells (Frick et al., 2016[6]). The association between autocrine and paracrine mechanism of CCL20 and patient’s prognosis has been reported in many cancer types (Rubie et al., 2010[23]; Coperchini et al., 2016[5]; Frick et al., 2016[6]; Kapur et al., 2016[9]; Marsigliante et al., 2016[16]; Wang et al., 2016[28]). In addition, metastasis in patients who received perioperative FOLFOX (the combination of oxaliplatin, fluorouracil and leucovorin) chemotherapy was associated with CCL20/CCR6 over-expression (Rubie et al., 2011[24]). Disruption of CCL20/CCR6 signaling would therefore be an alternative approach for cancer treatment. Interestingly, GSK3050002, a neutralizing antibody to CCL20, is available and a clinical trial has shown that it is safe to use in human subjects (Bouma et al., 2017[2]). Although CCR6-targeted treatments are still under development, it is another promising approach for modulating CCL20/CCR6 signaling (Robert et al., 2017[22]). Conceivably, CCL20/CCR6 targeting is a feasible approach that could be useful in the treatment of CCA.

## Conclusion

This study demonstrated the use of publicly available high throughput data for the global identification of biomarker for CCA. *CCL20*, which showed a consistent expression pattern across multiple datasets, was validated for its contribution to EMT-associated migration of CCA cell lines. This insight might be useful for the development of an alternative approach for the treatment of CCA. Modulation of CCL20/CCR6 signaling is very promising based on the availability and the favorable effect of the anti-CCL20 antibody. Targeting these molecules can help in controlling the migration of cancer cells, especially at the advanced stages of the disease, which is a major challenge for the management of CCA and other cancer types.

## Supplementary information

Supplementary information is available on EXCLI Journal website.

## Conflict of interest

The authors declare that they have no conflict of interest.

## Acknowledgements

We thank Dr. Aijaz Ahmad Malik for critical reading and proofreading the manuscript, Wisarut Lokunsup, Varat Laohaunya, and Wachiraporn Chaodorn for their contribution to the optimization of some experiments. This study was supported by grants A14/2560 from Mahidol University to PK and MRG6080286 from Thailand Research Fund to WC. The funder had no role in study design, data collection, and analysis, decision to publish and preparation of the manuscript.

## Supplementary Material

Supplementary material

Supplementary table 1

## Figures and Tables

**Table 1:**
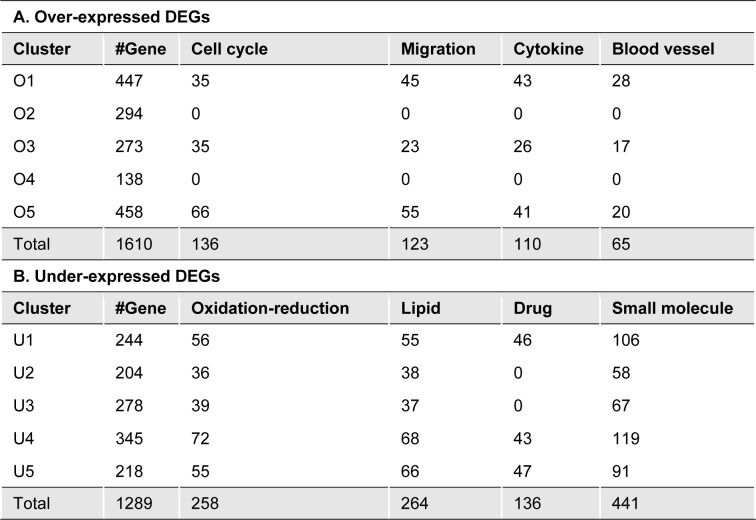
Size and the enriched biological processes in each subcluster
