# Supplementary material for: Common transcriptional programs and the role of chemokine (C-C motif) ligand 20 (CCL20) in cell migration of cholangiocarcinoma
Source: EXCLI J. 2020 Jan 20;19:154–66. doi: 10.17179/excli2019-1893 (PMC7068202; doi:10.17179/excli2019-1893)
Supplement: Supplementary material [file EXCLI-19-154-s-001.pdf]

EXCLI Journal 2020;19:154-166 – ISSN 1611-2156
Received: October 16, 2019, accepted: January 14, 2020, published: January 20, 2020

Supplemenatry information to:

COMMON TRANSCRIPTIONAL PROGRAMS AND THE ROLE OF CHEMOKINE (C-C MOTIF) LIGAND 20 (*CCL20*) IN CELL MIGRATION OF CHOLANGIOCARCINOMA

Hay Mar Win Maung[1], Waraporn Chan-On[2], Nawapol Kunkeaw[3], Prasong Khaenam[1*]

1 Center for Standardization and Product Validation, Faculty of Medical Technology, Mahidol University, Nakhon Pathom, 73170, Thailand
2 Center for Research and Innovation, Faculty of Medical Technology, Mahidol University, Nakhon Pathom, 73170, Thailand
3 Institute of Molecular Biosciences, Mahidol University, Nakhon Pathom, 73170, Thailand

* Corresponding author: Prasong Khaenam, Center for Standardization and Product Validation, Faculty of Medical Technology, Mahidol University, Nakhon Pathom, 73170, Thailand Phone: +66 2 441 4371 Ext. 2726 Fax: +66 2 412 4110;
E-mail: prasong.kha@mahidol.ac.th

http://dx.doi.org/10.17179/excli2019-1893

This is an Open Access article distributed under the terms of the Creative Commons Attribution License (http://creativecommons.org/licenses/by/4.0/).

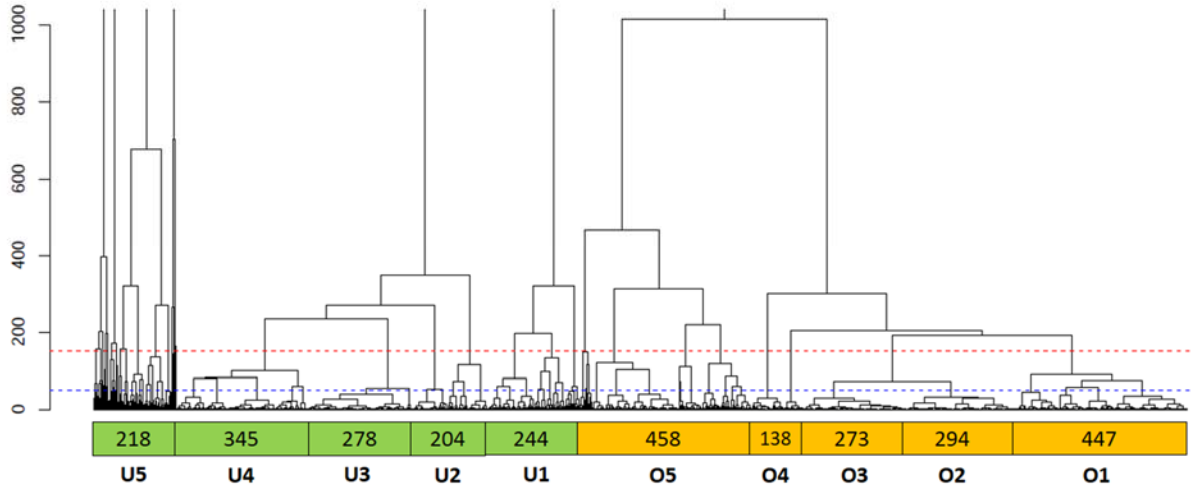

Supplementary Figure 1: Dendrogram of 2899 differentially expressed genes. Tree was cut mainly at the height of 150 for over-expressed DEGs (red dashed line) and 50 for under-expressed DEGs (blue dashed line). Original subcluster with small size (approximately 100 genes) was merged with nearest neighbors resulted in final subclusters as shown below the dendrogram. Size and subcluster labeling was shown at the bottom line.

EXCLI Journal 2020;19:154-166 – ISSN 1611-2156
Received: October 16, 2019, accepted: January 14, 2020, published: January 20, 2020

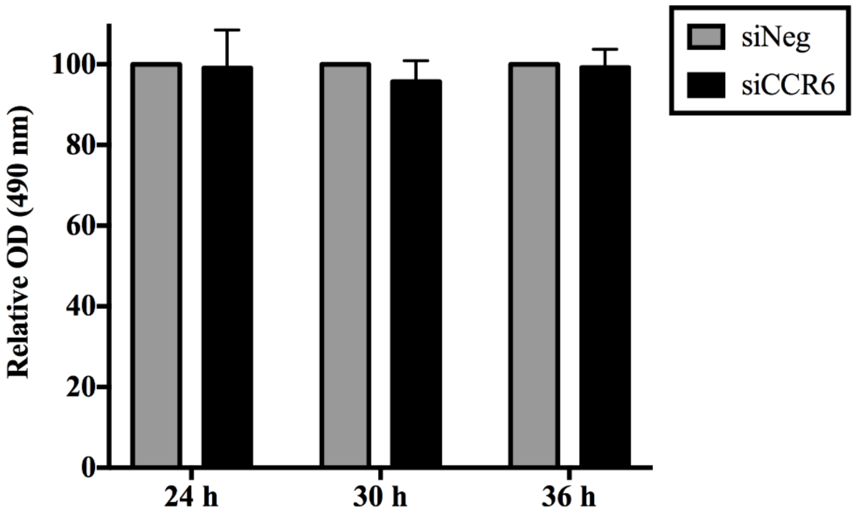

Supplementary Figure 2: The effect of siCCR6 on cell proliferation. Bar indicates mean ± SE from 3 independent sets of MTS assays.

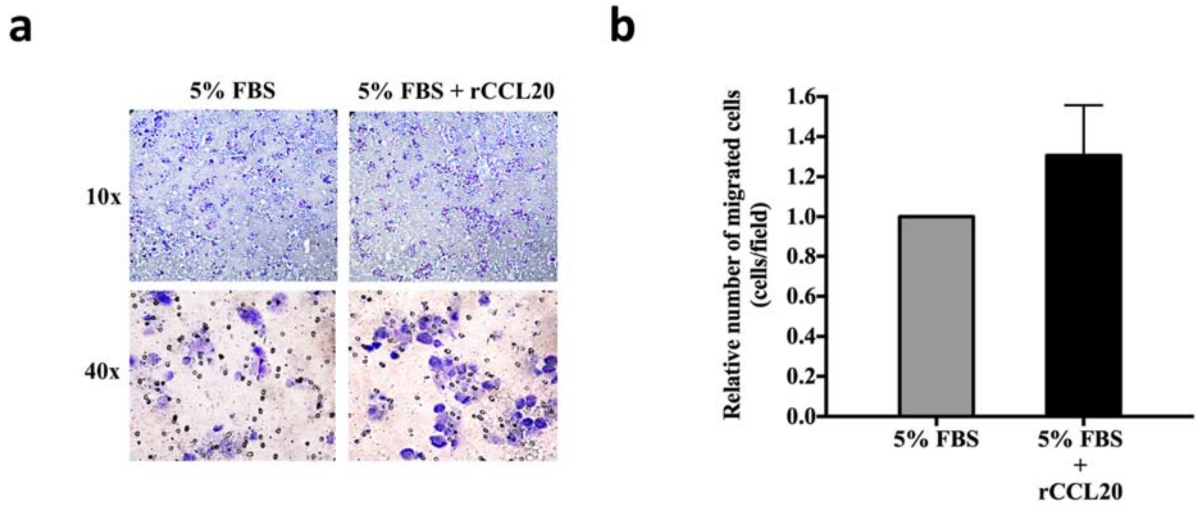

Supplementary Figure 3: The effect rCCL20 treatment in HuCCT1. **a**) The representative transwell images at 10X (top) and 40X (bottom) magnification for untreated and rCCL20 treated cells. **b**) Relative number of migrated cells
